# Seeing under the cover with a 3D U-Net: point cloud-based weight estimation of covered patients

**DOI:** 10.1007/s11548-021-02476-0

**Published:** 2021-08-21

**Authors:** Alexander Bigalke, Lasse Hansen, Jasper Diesel, Mattias P. Heinrich

**Affiliations:** 1 Institute of Medical Informatics, University of Lübeck, Ratzeburger Allee 160, 23538 Lübeck, Germany; 2 Drägerwerk AG & Co. KGaA, Moislinger Allee 53-55, 23558 Lübeck, Germany

**Keywords:** Clinical weight estimation, Deep learning, 3D U-Net, Point clouds, Covered patients

## Abstract

**Purpose:**

Body weight is a crucial parameter for patient-specific treatments, particularly in the context of proper drug dosage. Contactless weight estimation from visual sensor data constitutes a promising approach to overcome challenges arising in emergency situations. Machine learning-based methods have recently been shown to perform accurate weight estimation from point cloud data. The proposed methods, however, are designed for controlled conditions in terms of visibility and position of the patient, which limits their practical applicability. In this work, we aim to decouple accurate weight estimation from such specific conditions by predicting the weight of covered patients from voxelized point cloud data.

**Methods:**

We propose a novel deep learning framework, which comprises two 3D CNN modules solving the given task in two separate steps. First, we train a 3D U-Net to virtually uncover the patient, i.e. to predict the patient’s volumetric surface without a cover. Second, the patient’s weight is predicted from this 3D volume by means of a 3D CNN architecture, which we optimized for weight regression.

**Results:**

We evaluate our approach on a lying pose dataset (SLP) under two different cover conditions. The proposed framework considerably improves on the baseline model by up to ${16}{\%}$ and reduces the gap between the accuracy of weight estimates for covered and uncovered patients by up to ${52}{\%}$.

**Conclusion:**

We present a novel pipeline to estimate the weight of patients, which are covered by a blanket. Our approach relaxes the specific conditions that were required for accurate weight estimates by previous contactless methods and thus constitutes an important step towards fully automatic weight estimation in clinical practice.

## Introduction

Medical treatments often require the precise knowledge of a patient’s body weight, e.g. for patient-adapted drug dosing. In emergency situations, however, a straightforward assessment of the patient’s weight is often impossible. Unconsciousness of patients prevents a proper anamnesis, immobility impedes the usage of an ordinary scale, and bed scales are not always available. As a consequence, weight is often estimated by clinical staff although clinical studies have revealed the inaccuracy of these estimates [10, 21]. Several works suggest that there is a possibility to increase accuracy by inferring weight estimates from biometric measurements [5, 7, 19], but it is impractical to integrate the manual realization of these measurements into clinical routine. Instead, it is desirable to estimate the patient’s weight in a fully automatic and contactless way based on visual sensor data.

Pfitzner et al. [28] and our prior work [4] already demonstrated that machine learning-based methods are capable of deriving precise weight estimates of lying patients on the basis of point cloud data. Point clouds carry rich geometric information while preserving the patient’s data privacy [34] and are thus particularly suitable for the given problem. Whereas the proposed methods predict weight estimates with a promising accuracy, they involve a critical drawback: the methods are designed for and evaluated under highly controlled conditions. Patients are expected to be uncovered and in supine position in [4] and additionally even need to take a specific pose in [28].

In clinical practice, however, these specific demands are not always fulfilled. Patients take arbitrary poses and might be covered by a blanket. Especially the occlusion of the patient by a blanket considerably complicates the weight estimation problem and poses new challenges. First, it is no longer possible to identify a clear boundary between patient and mattress. Second, it is difficult to distinguish volume that actually belongs to the patient from volume belonging to the blanket and hollow space under the cover. As a consequence, existing methods are either no longer applicable at all [28] or suffer from a substantial degradation of accuracy [4]. Specifically, we evaluated our prior work [4] under occlusions by a blanket and observed an increase of the error of weight estimates by up to ${58}{\%}$. In practice, the predicted weight estimates will thus either be less accurate or clinical staff needs to manually remove the cover. Both options are unsatisfactory with regard to the intended fully automatic solution. Instead, an ideal weight estimation framework would provide reliable estimates independent of the presence of a cover. In this work, we aim to bring vision-based weight assessment closer to this level and address point cloud-based weight estimation of patients which are covered by a blanket.

### Related work

**General-purpose weight estimation** Body weight or body mass index estimation from full-body RGB, depth or RGB-D images has been addressed by numerous works, which predominantly rely on handcrafted geometric or biometric features [3, 13, 14, 16, 24, 37, 38]. In a common approach, the subject is segmented from the background, features are subsequently extracted from the silhouette, and weight regression is performed by a neural network or support vector regression [13, 16, 24, 38]. End-to-end learning of weight regression by means of deep convolutional neural networks has only been proposed by Nahavandi et al. [23] and Altinigne et al. [2], who utilize a U-Net [33] and a ResNet [12] architecture, respectively.

**Weight estimation in clinical settings** Most relevant to our work is weight estimation of patients lying in bed in a clinical environment. In an early work, Pirker et al. [29] generate a merged point cloud from depth information of eight stereo camera pairs, which are placed around the bed, and compute body part-specific volumes by fitting a parametric human 3D model to the cloud. More recently, Pfitzner et al. [26–28] predict the weight of a patient lying on a stretcher from a point cloud of a top-view depth camera. The authors start from a volume-based weight estimate in their initial work [26] and gradually include PCA-based features in [27] and contour-based features in [28], which are fed into a neural network for weight regression. Contrary to these feature-based methods, we proposed in our prior work [4] to use basis point sets (BPS) [30] for end-to-end learning of weight estimation from point clouds. All of these methods assume the patient to be fully visible and not to be covered by a blanket.

**Occlusion by a blanket** The occlusion of patients by a cover has been addressed by multiple works in the context of in-bed pose estimation. Achilles et al. [1] train and evaluate their pose estimation framework on depth maps with simulated blankets. Other approaches aim to see through the blanket by means of particular sensors, namely thermal cameras [18] or pressure mats [6]. In the context of weight estimation, however, such sensors appear less suitable than depth sensors, which capture richer geometric information. Multiple recent works estimate the patient’s pose and 3D shape under blanket occlusions from multi-modal input data by fitting or predicting the parameters of a 3D human mesh model [9, 15, 35, 42, 43]. Contrary to these works, our approach does not rely on a parametric model but explicitly addresses the occlusion problem in the input space.

**Deep learning from point clouds** Deep learning from unstructured 3D point cloud data has attracted much attention in recent years [11]. In a pioneering work, the PointNet architecture [31] applies a shared multi-layer perceptron to each input point individually and achieves a global permutation-invariant representation by max pooling. Subsequent works, such as PointNet++ [32] and Dynamic Graph CNNs [40], incorporate the structure of local neighbourhoods by means of hierarchical grouping and graph convolutions, respectively. In another line of work, point clouds are represented by 3D binary voxel grids, which are processed by 3D CNNs for shape classification [20, 41]. Beyond classification, the voxelized representation has been applied in the context of numerous other tasks, such as object detection [36] or pose estimation [22].

### Contribution

To our knowledge, this is the first work to learn weight prediction of covered patients. In the light of the identified challenges in this setting, we regard weight estimation and occlusion by a blanket as two separate, independent problems and consequently propose a two-step solution. In a first step, we virtually remove the blanket by predicting the patient’s shape without a blanket. For this purpose, we resort to a voxelized representation of point clouds and train a 3D U-Net [8] to accomplish the task. This step is independent of the weight estimation problem and can be used as a pre-processing step for other tasks as well. In a second step, the actual weight regression is performed by a customized 3D CNN, which no longer needs to overcome the occlusion by a blanket. Thus, our proposed method essentially simplifies the overall problem and, as a beneficial by-product, provides a high degree of interpretability owing to the intermediate visualization of the uncovered patient.

The main contributions of this work can be summarized as follows:

- We introduce a novel two-stage pipeline of two 3D CNNs to predict the weight of covered patients from voxelized point cloud data.
- We propose to virtually uncover the patients to simplify the weight estimation problem and demonstrate the capability of a 3D U-Net to solve this task.

## Methods

In this section, we present our approach for weight estimation of covered patients from point cloud data. We initially formalize the problem set-up, subsequently give an overview of the proposed framework and finally present its individual components in detail.

### Problem set-up

Our goal is to develop a method that takes a 3D point cloud ${\varvec{X}}^c\in \mathbb {R}^{N\times 3}$, which shows a covered patient lying in bed, as input and predicts the weight *y* of the patient. For this purpose, we assume access to a training dataset $\mathcal {T}=\{({\varvec{X}}_i^{c}, {\varvec{X}}_i^{\smallsetminus c},y_i)\}_i$. It consists of pairwise point clouds ${\varvec{X}}_i^{c}, {\varvec{X}}_i^{\smallsetminus c}$, which show a patient in unchanged pose with (${\varvec{X}}_i^{c}$) and without (${\varvec{X}}_i^{\smallsetminus c}$) a cover, respectively, together with the ground truth weight $y_i$ of the patient.

### Framework

**Fig. 1:**
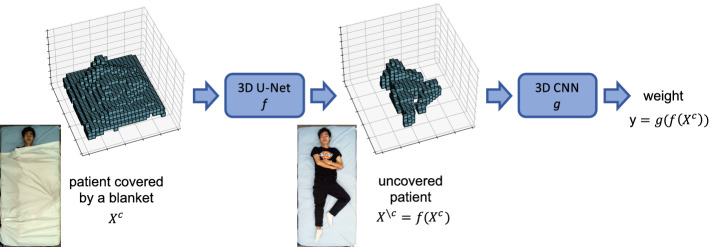
Overview of our proposed two-stage pipeline for weight estimation of covered patients. Based on the voxelized input point cloud, we virtually uncover the patient with a 3D U-Net and perform the actual weight estimation based on the uncovered volume with a 3D CNN. Colour images are only shown for better visualization and are not used in the pipeline

An overview of our proposed framework is visualized in Fig. 1. The core idea of our approach is to decouple the weight estimation problem itself from the occlusion problem caused by the blanket. To this end, we break the overall task down into two independent sub-problems, which are solved in two successive steps. In step one, we virtually remove the cover from the patient. By leveraging pairwise point clouds from $\mathcal {T}$, we learn to predict the patient’s shape without a cover. This substantially simplifies the actual weight estimation performed in step two. The weight estimation problem is no longer complicated by a blanket and can thus be solved as for an uncovered patient.

In step one, we formally aim to learn a mapping from ${\varvec{X}}^{c}$ to ${\varvec{X}}^{{\smallsetminus } c}$. Due to the inherent lack of point correspondences between two point clouds, however, it poses several technical challenges to properly define this problem for raw point clouds. Therefore, we resort to a voxelized representation such that two point clouds are naturally aligned. To voxelize a point cloud ${\varvec{X}}$, a fixed-size cuboid volume around the cloud is discretized into a set of equally sized voxels, and a voxel is assigned the value 1 if it contains at least one point of the cloud and 0 otherwise. The resulting representation constitutes a binary 3D volume ${\varvec{X}}^{vox}\in \{0,1\}^{h\times w\times d}$, where *h*, *w* and *d* denote the number of voxels in *x*-, *y*-, and *z*-direction, respectively. For ease of notation, we will omit the superscript from now on, and ${\varvec{X}}$ refers to the voxelized representation of a point cloud.

This representation enables us to formalize the task in step 1. Specifically, we intend to learn a function *f* with parameters $\varvec{\theta }_f$ that takes the volume of a covered patient ${\varvec{X}}^{c}$ as input and outputs the volume of the uncovered patient ${\varvec{X}}^{{\smallsetminus } c}$, i.e. $f({\varvec{X}}^{c};\varvec{\theta }_f)={\varvec{X}}^{{\smallsetminus } c}$. In our pipeline, we implement *f* as a 3D U-Net [8] as detailed in Sect. 3D U-Net and optimize its parameters by minimizing the cross-entropy loss

1 $$\begin{aligned} \mathcal {L}(\varvec{\theta }_f;\mathcal {T})=\sum _{({\varvec{X}}_i^{c},{\varvec{X}}_i^{\smallsetminus c})\in \mathcal {T}}\mathrm {CE}(f({\varvec{X}}_i^{c};\varvec{\theta }_f),{\varvec{X}}_i^{\smallsetminus c}) \end{aligned}$$

with respect to $\varvec{\theta }_f$. Here, $\mathrm {CE}(\cdot ,\cdot )$ denotes the element-wise binary cross-entropy loss function.

Once we have learnt to uncover the patient in step 1, we subsequently learn a function *g* with parameters $\varvec{\theta }_g$, which takes the volume of the uncovered patient predicted by *f*, namely $f({\varvec{X}}^{c};\varvec{\theta }_f)$, as input and outputs the patient’s weight *y*, i.e. $y=g(f({\varvec{X}}^{c};\varvec{\theta }_f);\varvec{\theta }_g)$. We implement *g* as a 3D CNN introduced in Sect. 3D CNN for weight regression. The optimization is performed by minimizing the mean-squared error loss

2 $$\begin{aligned} \mathcal {L}(\varvec{\theta }_f, \varvec{\theta }_g; \mathcal {T})=\sum _{({\varvec{X}}_i^{c}, y_i)\in \mathcal {T}}\Big [g(f({\varvec{X}}_i^{c};\varvec{\theta }_f);\varvec{\theta }_g)-y_i\Big ]^2 \end{aligned}$$

with respect to $\varvec{\theta }_g$ while keeping $\varvec{\theta }_f$ fixed.

### 3D U-Net

The architecture of the 3D U-Net strictly follows the original implementation in [8]. In short, the 3D U-Net comprises a contracting encoder path and an expanding decoder path, both including four levels of different resolutions. In the encoder path, features are extracted by means of 3D convolutions and downsampling is realized by max pooling operations. In the decoder path, low-resolution features are gradually upsampled by transposed convolutions and combined with high-resolution features of equal resolution from the encoder path. This is realized by skip connections and subsequent 3D convolutions, which merge the features.

### 3D CNN for weight regression

**Fig. 2:**
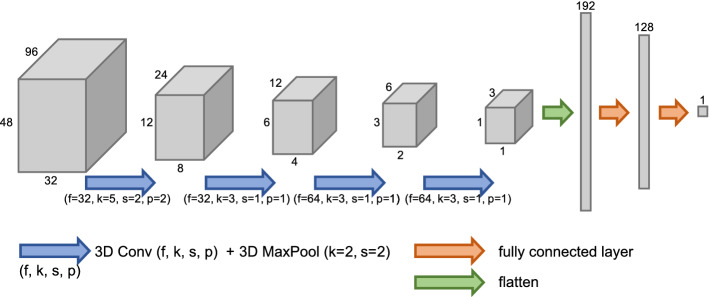
Visualization of our proposed 3D CNN for weight estimation from a 3D volume. 3D convolutions are characterized by the number of output feature channels *f*, kernel size *k*, stride *s* and padding *p*. *k*, *s* and *p* are identical in all spatial dimensions

The architecture of our proposed 3D CNN for weight regression from 3D volumes is illustrated in Fig. 2. During optimization of the architecture, we found it crucial to downsample the input volume to very low resolution before final weight regression with the fully connected network heads. To realize this, the architecture starts with a 3D convolution with a kernel size of $5\times 5\times 5$ and a stride of 2, followed by a max pooling operation with kernel size $2\times 2\times 2$. Subsequently, the resulting feature map is alternatingly processed by 3D convolutions with kernel size $3\times 3\times 3$ and stride 1, and max pooling operations with kernel size $2\times 2\times 2$, which further reduce the spatial resolution. That way, the input volume is downsampled by a factor of $2^5$ in each spatial dimension. At the same time, the number of feature channels is gradually increased to 64. Each convolutional layer is followed by a batch normalization layer and a ReLU nonlinearity. After the last convolutional layer, the feature maps are flattened and forwarded by the fully connected network heads. These consist of a fully connected layer with 128 neurons, followed by ReLU nonlinearity, dropout (*p* = 0.8) and the output neuron with linear activation.

## Experiments and results

Dataset We evaluate our method on the SLP dataset [17]. The dataset consists of depth frames of 109 subjects, which take 45 different poses while lying in bed in supine and lateral (left and right) positions. For each pose, three nearly identical depth frames are taken, which only differ in terms of the cover condition (no cover, thin cover, thick cover) and are thus ideal for learning the virtual removal of a blanket. Covers have a thickness of around 1 and ${3}{\hbox { mm}}$, respectively. For each frame, we detect all pixels belonging to bed and patient with the help of depth thresholding and clustering and transform these pixels to a point cloud using the internal camera parameters. Body weights of the subjects range from 43.7 to ${105.1}{\hbox { kg}}$ and exhibit a mean of ${68.0}{\hbox { kg}}$ and a standard deviation of ${12.7}{\hbox { kg}}$.

**Table 1:** Weight estimation results on the 42 subjects from the laboratory setting of the SLP dataset

Method	No cover		Thin cover		Thick cover	
	Supine	Lateral	Supine	Lateral	Supine	Lateral
PointNet [31]	$4.66\pm 0.23$	$4.13\pm 0.25$	$5.97\pm 0.12$	$5.92\pm 0.06$	$6.14\pm 0.11$	$6.00\pm 0.04$
BPS [4, 30]	$4.33\pm 0.18$	$4.07\pm 0.19$	$6.13\pm 0.11$	$6.34\pm 0.17$	$6.68\pm 0.13$	$6.64\pm 0.17$
3D CNN	$\varvec{3.86\pm 0.12}$	$\varvec{3.80\pm 0.05}$	$5.36\pm 0.06$	$5.28\pm 0.06$	$5.56\pm 0.07$	$5.45\pm 0.04$
3D U-Net + 3D CNN (weight regr. only e2e)	–	–	$5.25\pm 0.03$	$5.17\pm 0.04$	$5.44\pm 0.07$	$5.24\pm 0.05$
3D U-Net + 3D CNN (ours)	–	–	$\varvec{4.61\pm 0.06}$	$\varvec{4.51\pm 0.04}$	$\varvec{4.71\pm 0.13}$	$\varvec{4.54\pm 0.10}$

We compare the MAE, measured in kg, of several baseline methods to our proposed framework

The dataset includes two different set-ups. One hundred and two subjects were recorded in a laboratory setting, and the remaining seven subjects were recorded in a simulated hospital room. The two settings differ in terms of the used beds, mattresses, sheets, blankets, and sensor-to-bed distances, resulting in a substantial domain shift. We conduct the main experiments with the 102 subjects, training the model on the first 60 subjects and reporting results for the remaining 42 subjects. The seven subjects recorded in the hospital room are used for cross-domain evaluation. Generally, the model is jointly trained under both cover conditions (thin cover, thick cover) and positions (supine, lateral), while evaluation is performed for each cover type and position separately.

Implementation Details We implement our proposed framework in PyTorch [25]. Network parameters are optimized with the ADAM optimizer. The initial learning rate is set to 0.001, and we use a batch size of 16. The 3D U-Net is trained for 50 epochs, whereby the learning rate is divided by 10 after 30 epochs. The 3D CNN for weight regression is trained for 120 epochs, whereby the learning rate is divided by 10 at epoch 60 and 100. For voxelization of the raw point clouds, we discretize a cuboid volume of size ${1.7}{\hbox { m}}\times {2.4}{\hbox { m}}\times {0.7}{\hbox { m}}$ into $48\times 96\times 32$ voxels with edge lengths of ${3.5}{\hbox { cm}}\times {2.5}{\hbox { cm}}\times {2.2}{\hbox { cm}}$. The size of the cuboid volume has been chosen such that it covers all mean-centred clouds from the training set. When training the U-Net in step 1, we pre-process the target point cloud of the uncovered patient before voxelization to segment the patient from the bed [4].

Baseline Methods We consider two baseline methods for weight estimation from covered patients. First, we train the plain 3D CNN without preceding U-Net for weight regression. Second, for a fair comparison regarding the number of model parameters, we train the composition of 3D U-Net and 3D CNN for weight regression in an end-to-end fashion without minimizing the intermediate loss in Eq. (1). As an upper bound, we train the plain 3D CNN to predict the weight of uncovered patients. To further investigate the effect of occlusions by a blanket on weight estimation performance, we additionally learn weight estimation of both covered and uncovered patients with a PointNet architecture [31] and the BPS-based fully connected network from [4], which operate on raw point cloud data instead of a voxel-based representation.

Evaluation As error metric, we use the mean absolute error (MAE) of the predicted weight estimates on the test set. Each experiment is repeated 5 times, and we report mean and standard deviation of the MAE.

### Results

Quantitative results of our main experiments are presented in Table 1 and reveal four major insights.

First, we note that weight estimates in supine and lateral position have a similar accuracy under all cover conditions and for all models, whereby estimates in lateral position are in most cases slightly better.

Second, we observe that occlusions by both a thin and a thick cover lead to a substantial degradation of the performance of PointNet, BPS and 3D CNN. As expected, the performance for the thick cover is consistently slightly worse than for the thin cover. As an interesting side note, we notice that the voxel-based 3D CNN clearly outperforms both point cloud-based approaches under all three cover conditions. But even for the 3D CNN, we observe a relative increase in MAE of ${39}{\%}$ for the thin cover and of ${44}{\%}$ for the thick cover (averaged over supine and lateral position). This confirms the need for weight estimation methods that explicitly address the specific challenges caused by a partial covering of the patient by a blanket.

**Fig. 3:**
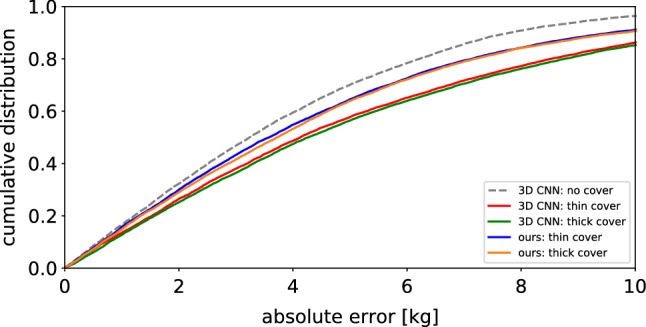
Cumulative distribution of weight estimation errors for our method and the 3D CNN baseline. Our method clearly improves on the baseline model under both cover conditions and reduces the gap to weight estimates from uncovered patients

**Fig. 4:**
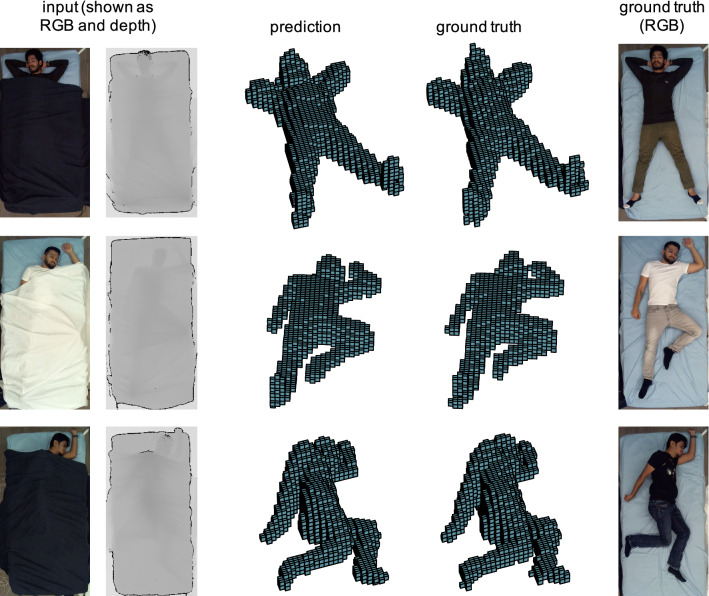
Qualitative results of the ablation study on three samples from the SLP dataset. From left to right, each row shows RGB and depth image of the corresponding input volume, the prediction by our method, ground truth and the RGB image of the ground-truth scene. RGB and depth images are shown for better visualization, while our framework processes and outputs volumetric representations

**Table 2:** Quantitative results of the ablation experiment on the 42 subjects from the laboratory setting of the SLP dataset

Method	Metric	Thin cover		Thick cover	
		Supine	Lateral	Supine	Lateral
Initial	Dice	31.0	28.4	30.7	27.7
3D U-Net		$76.1\pm 0.1$	$75.0\pm 0.2$	$76.1\pm 0.1$	$73.9\pm 0.1$
Initial	Surface distance	12.1	12.4	11.8	12.6
3D U-Net		$5.0\pm 0.1$	$4.9\pm 0.1$	$5.0\pm 0.1$	$5.1\pm 0.1$

We show the Dice overlap and the average surface distance in mm between the volume of the uncovered patient predicted by the 3D U-Net and the corresponding ground truth. For reference, we report the initial Dice overlap and surface distance between uncovered and covered patient volume

Third and most importantly, it can be seen that our proposed method successfully addresses these challenges and considerably outperforms the baseline models under both cover conditions. Specifically, again averaged over supine and lateral position, the MAE is reduced by ${14.3}{\%}$ for the thin cover and by ${16.0}{\%}$ for the thick cover with respect to the baseline 3D CNN. Moreover, the gap between weight estimates with and without a cover achieved by the 3D CNN is reduced by ${51.0}{\%}$ for the thin cover and by ${52.5}{\%}$ for the thick cover.

Fourth, we observe that the composition of 3D U-Net and 3D CNN, exclusively trained for weight regression in an end-to-end fashion, only slightly improves on the performance of the plain 3D CNN. We deduce that the superiority of our method is merely to a small extent due to increased model capacity. Rather, it is crucial to explicitly learn to virtually uncover the patient.

Finally, we provide a more detailed comparison of our method and the 3D CNN by plotting the cumulative distributions of absolute errors in Fig. 3. The observable trends are in line with the findings discussed above.

### Ablation experiment

In the ablation experiment, we intend to assess the capability of the U-Net to uncover a patient in a more direct way. For quantitative evaluation, we compute the average surface distance from the target volumes ${\varvec{X}}^{{\smallsetminus } c}$ to the outputs of the U-Net $f({\varvec{X}}^{c})$ as well as the Dice overlap between both volumes. For reference, we report the initial average surface distance and the intial Dice overlap between ${\varvec{X}}^{{\smallsetminus } c}$ and ${\varvec{X}}^c$. Results of the experiment are shown in Table 2. Under both cover conditions and positions the U-Net more than doubles the initial Dice overlap and more than halves the initial surface distance. This demonstrates its capability to virtually uncover the patient with an adequate accuracy.

Regarding the Dice score, we observe a small gap between supine and lateral positions. This gap is most likely not due to less accurate predictions but due to the sensitivity of the Dice score to the size of ground-truth volumes. Ground-truth volumes in lateral positions are represented by less voxels than in supine positions such that errors have a larger negative impact on the Dice score. Referring to the average surface distance, which is less sensitive to the size of objects, the score is similar for both positions.

Qualitative results of the ablation study are presented in Fig. 4 and demonstrate that the predictions by the 3D U-Net are visually compelling as well. 3D volumes of the subjects are precisely recovered under varying poses and even hollow space under the blanket is largely correctly classified as not being part of the human body.

### Cover detection for full automation

In practical applications, it is desirable to estimate the weight of both covered and uncovered patients with a single fully automatic pipeline. For this purpose, we initially need to classify whether patients are covered or uncovered. Subsequently, the weight of uncovered patients can be estimated by the 3D CNN, while the weight of covered patients is assessed by our entire framework of 3D U-Net and 3D CNN.

To automate cover detection, we train the baseline 3D CNN as a binary classifier. The network is trained for 10 epochs with an initial learning rate of 0.001, which is divided by 10 after 5 and 8 epochs. The 3D CNN achieves a classification accuracy of ${100.0}{\%}$. Thus, subsequent weight estimation of a patient is virtually always performed by the appropriate framework and the accuracies reported in Table 1 remain unchanged in a fully automatic pipeline.

### Cross-domain evaluation

To examine the cross-domain robustness of our method, we evaluate all models from Table 1 (trained on the 60 subjects from the laboratory setting) on the seven subjects from the simulated hospital room. Due to the substantial domain shift between training and test data, this is a challenging setting. Specifically, the varied sensor-to-bed distance leads to a different distribution of points in 3D space, the change of the bed alters the geometry of the entire scene, and mattresses and bed sheets might differ in terms of firmness and flexibility. Quantitative results of the experiment are shown in Table 3. On the one hand, our proposed method outperforms all baseline methods even in this more complicated setting. Compared to the baseline 3D CNN, the MAE is reduced by ${19.5}{\%}$ for the thin cover and by ${13.6}{\%}$ for the thick cover. This indicates that our two-step solution is more robust than end-to-end approaches. On the other hand, however, we observe that all methods exhibit a severe performance drop under all cover conditions compared to the in-domain evaluation (Table 1). For instance, the baseline 3D CNN deteriorates by ${73}{\%}$, ${65}{\%}$ and ${79}{\%}$ for no cover, thin cover and thick cover, respectively, and our method degrades by ${55}{\%}$ for the thin cover and by ${84}{\%}$ for the thick cover. We conclude that the domain shift is a serious problem that needs to be addressed by methods from domain adaptation [39]. However, this is beyond the scope of this work and is left to future research. Our results constitute an initial baseline for such work.

**Table 3:** Results of the cross-domain evaluation on the 7 subjects from the hospital room of the SLP dataset. Bold values highlight the best results with the lowest MAE

Method	No cover	Thin cover	Thick cover
PointNet [31]	$6.99\pm 0.34$	$9.94\pm 0.80$	$10.91\pm 0.63$
BPS [4, 30]	$6.69\pm 0.57$	$9.57\pm 1.21$	$12.41\pm 2.41$
3D CNN	$\varvec{6.62\pm 0.39}$	$8.76\pm 0.54$	$9.85\pm 0.47$
3D U-Net + 3D CNN (weight regr. only e2e)	–	$8.93\pm 0.61$	$9.75\pm 0.40$
3D U-Net + 3D CNN (ours)	–	$\varvec{7.05\pm 0.20}$	$\varvec{8.51\pm 0.40}$

We compare the MAE, measured in kg and averaged over supine and lateral positions, of several baseline methods to our proposed framework

## Discussion and conclusion

We proposed a novel framework, consisting of a 3D U-Net and a 3D CNN, for weight estimation of covered patients from voxelized point clouds. In our experiments on the SLP dataset, we demonstrated that the 3D U-Net is capable of virtually uncovering a patient and to thus simplify the subsequent weight regression with the 3D CNN. Specifically, our method improved the weight estimation performance compared to baseline methods by up to ${16}{\%}$ and reduced the gap to weight estimates of uncovered patients by up to ${52}{\%}$. Even in presence of a thick cover, our method achieves a higher accuracy (MAE = 4.62 kg, corresponding to a mean relative error (MRE) of ${7.0}{\%}$) than estimates by clinical staff, which exhibit MREs of 8.1–8.4% in [10] and of 7.7–11.0% in [21]. The accuracy of our method can even further be improved to an MAE of 3.8 kg and an MRE of ${5.7}{\%}$ by statistical averaging over multiple weight estimates for the same subject from different frames with varying poses as in [4]. Altogether, our work constitutes an important step towards fully automatic weight estimation, which should ideally provide reliable weight estimates independent of any specific conditions. However, the occlusion of a patient by a cover is only one among multiple possible challenges, which might occur in clinical practice. Another important problem, for instance, consists in the presence of a domain shift between training and test data, which lead to a substantial performance drop in our cross-domain experiment. To improve generalization to diverse settings (different room set-ups, beds, mattresses, viewpoints, etc.), future work could incorporate techniques from domain adaptation [39] or domain generalization [44] into the weight estimation framework.

Beyond the specific task of weight estimation, we believe that our approach to virtually uncover the patient constitutes a valuable tool for medical computer vision in general. Since the method is independent of the weight estimation problem, it can be seen as a generic pre-processing step with the potential to simplify any task that needs to overcome occlusions by a blanket. In particular, the integration of the approach into existing frameworks for in-bed pose and shape estimation [1, 35] appears promising and is of high interest for future work.

## Data Availability

The used dataset is available upon request from the authors of [17].

## Declarations

### Code availability

Source code is available at https://github.com/multimodallearning/weight-estimation-under-cover
